# *In situ* detection of fruit spoilage based on volatile compounds using the mid-infrared fiber-optic evanescent wave spectroscopy

**DOI:** 10.3389/fpls.2022.991883

**Published:** 2022-10-11

**Authors:** Yunhai Zhou, Yifan Gu, Rui Guo, Leizi Jiao, Ke Wang, Qingzhen Zhu, Daming Dong

**Affiliations:** ^1^ School of Agricultural Engineering, Jiangsu University, Zhenjiang, China; ^2^ National Research Center of Intelligent Equipment for Agriculture, Beijing Academy of Agriculture and Forestry Sciences, Beijing, China

**Keywords:** *in situ* detection, fruit spoilage, volatile compounds, ethanol, FOEW spectroscopy, FTIR

## Abstract

Volatile compounds such as ethanol released from fruit can be rapidly detected using Fourier Transform Infrared spectroscopy based on a long-path gas cell. However, this method relies on a long optical path length and requires pumping fruit volatiles into the gas cell. This can lead to the volatile compounds being contaminated and not detectable *in situ*. Fiber optic evanescent wave spectroscopy (FOEW) is not influenced by the path length so can detect materials (solid, liquid and gas phase) rapidly *in situ*, using only a few millimeters of optical fiber. In the present study, a spiral silver halide FOEW sensor with a length of approximately 21 mm was used to replace a long-path gas cell to explore the feasibility of identifying volatile compounds released from grapes *in situ*. The absorption peaks of ethanol in the volatile compounds were clearly found in the FOEW spectra and their intensity gradually increased as the storage time of the grapes increased. PCA analysis of these spectra showed clear clustering at different storage times (1-3, 4-5 and 6-7 d), revealing that the concentration of the ethanol released from the grapes changed significantly with time. The qualitative model established by PLS-DA algorithm could accurately classify grape samples as “Fresh,” “Slight spoilage,” or “Severe spoilage”. The accuracy of the calibration and validation sets both were 100.00%. These changes can therefore be used for rapidly identifying fruit deterioration. Compared with the method used in a previous study by the authors, this method avoids using a pumping process and can thus identify volatile compounds and hence monitor deterioration *in situ* and on-line by placing a very short optical fiber near the fruit.

## Graphical Abstract

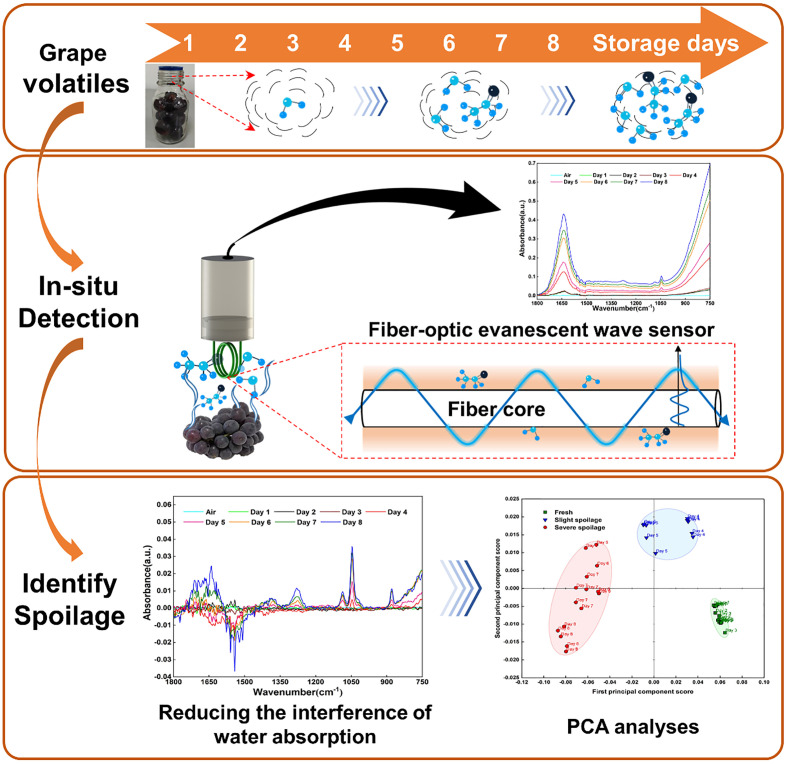

## Highlights

- Sensitive detection of volatile compounds using a FOEW sensor to replace a long-path gas cell.
- Sensitive detection of volatile compounds can be achieved independently of long optic path.
- *In situ* and on-line detection of ethanol by placing a short FOEW sensor near the fruits.
- Fruit spoilage can be identified *in situ* by a FOEW sensor and FTIR spectrometer.

## 1 Introduction

Fruit is essential for human health with its high contents of vitamins, minerals, antioxidants and many phytonutrients (Rice-Evans and Miller, 1995). However, fruit is frequently transported over long distances, allowing time for it to spoil easily, with the consequent food safety concerns and heavy economic losses. It has been estimated that approximately 20% of all fruit produced for human consumption is lost each year because of spoilage (Jahun et al., 2021). Therefore, the rapid identification of fruit spoilage *in situ* is of great significance for ensuring food safety, reducing economic losses and improving human living standards. The volatile compounds released from fruit reflect their particular status during storage. Several studies have reported that the concentrations of volatile compounds emitted from fruit, such as alcohols, esters, terpenes and ethylene, are closely related to their quality (Zhu et al., 2018a; Cai et al., 2019; Wu et al., 2020). Thus, the qualitative and quantitative analyses of these volatile compounds are critical for rapidly assessing the quality of fruit.

Gas Chromatography (GC) and Gas Chromatography Mass Spectrometry (GC-MS) are commonly used in most research studies for accurately detecting volatiles released from fruit (Sánchez-Palomo et al., 2005; Giannetti et al., 2017; Zhu et al., 2018b). These methods detect differences in the adsorption capacity of adsorbents in the chromatographic column for the qualitative and quantitative detection of fruit volatiles. Although these laboratory physical and chemical analysis methods are sensitive and accurate, they require complex sampling, preparation and analysis processes. This method requires time and effort compared to spectroscopy, which does not necessitate sample preparation. For fruit volatiles, a rapid detection method is required. Stimulated by this demand, Proton Transfer Reaction Mass Spectrometry (PTR-MS) (Hewitt et al., 2003; Franke and Beauchamp, 2016), which can be linked to a quadrupole (PTR-(Quad)MS) or time-of-flight mass spectrometer (PTR-TOF-MS) (Liu et al., 2018), has been used to detect fruit volatiles on-line (Cappellin et al., 2012; Li et al., 2021). However, the equipment must be calibrated with a standard gas for quantitative analysis, and requires several minutes to stabilize for reagent ion switching. It is also important to note that PTR-MS only provides molecular weight information (mass-to-charge ratio) on the volatile compounds and cannot be used for the qualitative analysis of isomers. It can not achieve the recognition accurate of spectroscopy. Compared with these complex laboratory analysis methods, an electronic nose (E-nose) based on electrochemical reactions has the advantages of low cost, portability and ease of use. It can quickly generate a superimposed signal response of the volatile compounds almost in real time. Some studies have used the E-nose to determine the stages of fruit spoilage and assess the physical and chemical properties of fruit (Chen et al., 2018; Tatli et al., 2021). However, the data generated by the E-nose is complex and must be processed by multivariate statistical methods for their accurate interpretation, compared with spectroscopy, its reproducibility, resolution and robustness still needing to be resolved.

Based on the specific excitation of rotation and vibrational transitions of most molecular compounds, mid-infrared (MIR) spectroscopy provides quantitative information on the unique chemical and structural properties of molecules and has been widely used in medicine (Tiernan et al., 2020), agriculture (Volkov et al., 2021) and general industry as a rapid and sensitive technique for obtaining molecular fingerprints (Ke et al., 2018). Most volatile compounds released from food have specific infrared absorption characteristics and can be analyzed qualitatively and quantitatively using these specific infrared absorption peaks, with many studies demonstrating the feasibility of this technique (Cubero-Leon et al., 2014; Jahromi et al., 2021). Since 2013, this research group has been studying MIR spectroscopy methods to detect the volatile compounds released from food during storage as it matures and spoils (Dong et al., 2019). The volatile compounds released from fruit, such as grapes, strawberries and mangoes, have been measured using Fourier Transform Infrared (FTIR) spectrometry using a long-path gas cell for predicting the stages of fruit spoilage and ripeness (Dong et al., 2013; Jiao et al., 2017; Jiao et al., 2019). This method is clearly useful but depends on the optical path length. A large long-path gas cell (up to 20 m) was necessary for the sensitive detection of volatile compounds. This method required a blower to pump the volatile compounds into the gas cell, leading to their possible contamination and thus not detectable *in situ*.

Fiber optic evanescent wave (FOEW) sensing technology was first developed by Paul and Kychakoff (Paul and Kychakoff, 1987). Its mode of detection is based on the interaction between evanescent waves and the absorbing medium surrounding the fiber core (Alvarez-ordóñez et al., 2011; Blum and John, 2012; Sharma et al., 2019). Using only a few centimeters of optical fiber, it can detect volatile compounds in solution (Lu et al., 2016; Memon et al., 2017; Dettenrieder et al., 2019; Jiao et al., 2020), *in situ* and on-line, and also where limited space is available. However, the density of gas is low compared with that of a liquid, meaning that the number of molecules on the optical fiber core surface is low at any given time. Therefore, detecting volatile compounds in air using MIR FOEW spectroscopy is not common and remains a challenging technique. Recently, using a single mode chalcogenide glass optical waveguide, Jin et al. (2019) have verified the feasibility of using evanescent wave spectroscopy for selectively detecting ethanol in air (Jin et al., 2019), which provided a basis for detecting fruit volatiles in air based on FOEW spectroscopy as used in the present study. To the best of our knowledge, there have been no reports on using FOEW spectroscopy to detect fruit volatiles, and its feasibility needs to be verified.

The present study, based on previous studies, aims to use a spiral silver halide FOEW sensor with a length of approximately 21 mm to replace the long-path gas cell to check the feasibility of identifying the ethanol in volatile compounds released from fruit *in situ*. The major advantages and innovations of the proposed method are that it requires no sample pretreatment, the identification *in situ* does not require the volatile compounds to be pumped into the gas cell so does not rely on a long optical path length, thus allowing it to be used in practical food storage situations.

## 2 Materials and methods

### 2.1 Materials

Fresh grapes (Kyoho, Tianjin, China) were purchased from a local supermarket in Beijing, China. These grapes were carefully separated one by one to ensure that their surfaces were free of mechanical damage. The experiment used 5 independent samples of grapes, each consisting of 150 g. Five glass bottles were cleaned and pasteurized to store the grape samples to collect their volatile compounds during storage. The same glass bottles were later used to hold different concentrations of ethanol standard gases. Plastic wrap was used to seal these bottles and contain the volatiles. Plastic tubes with a volume of 60 ml were used to store ethanol and deionized solutions to produce the headspace volatile compounds for analysis.

### 2.2 Instruments

**Figure 1:**
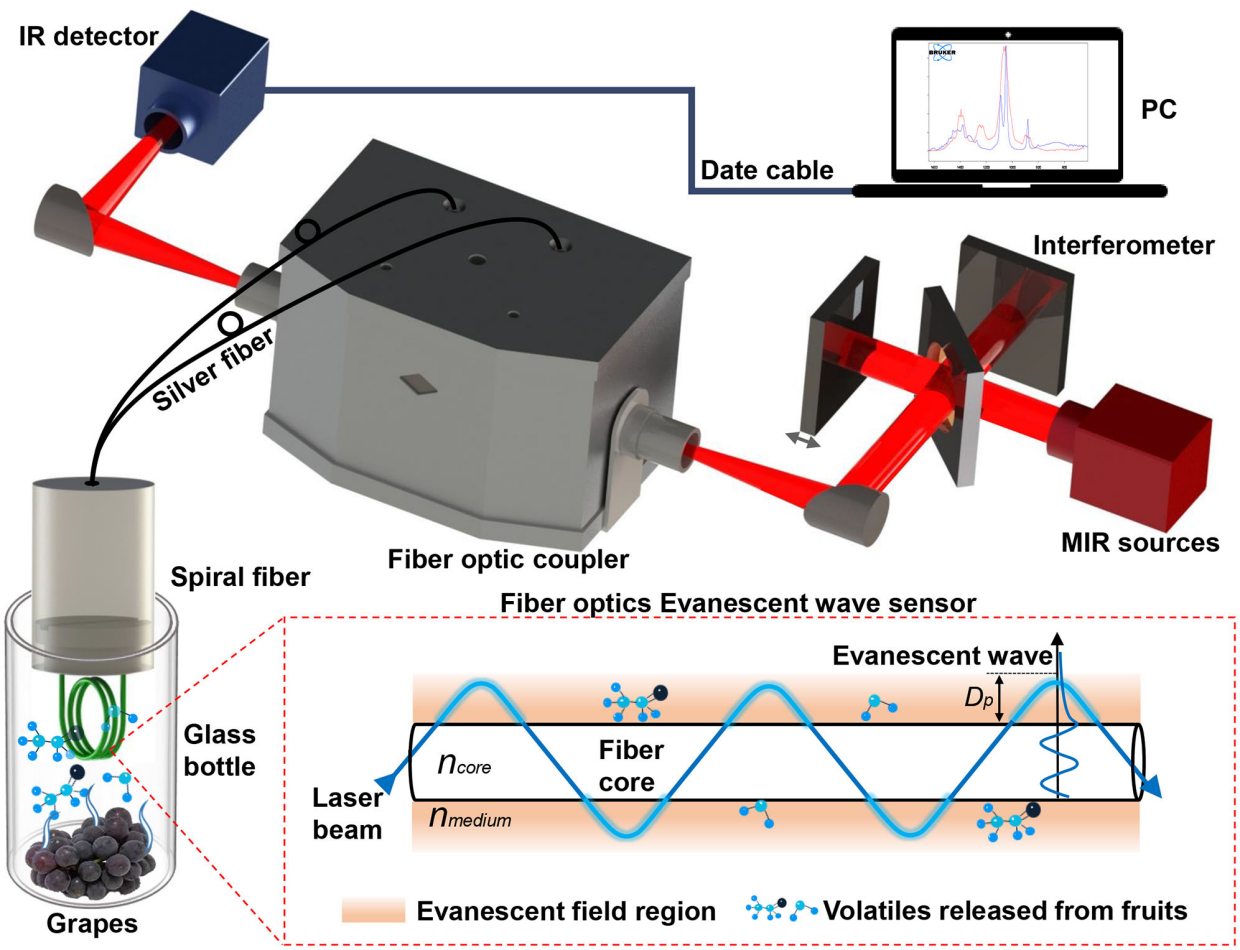
Schematic diagram for detecting volatile compounds released from grapes *in situ* based on the MIR FOEW spectrum.

The schematic diagram for detecting the volatile compounds of grapes based on the FOEW sensor is shown in **Figure 1**. The FTIR spectrometer (Vertex 70, Bruker Ltd., Karlsruhe, Germany) was equipped with a MIR source, an interferometer and an infrared (IR) detector. It could obtain infrared spectra in the range of 4000–600 cm^-1^ with high levels of stability and sensitivity by cooling the detector with liquid nitrogen. The spectral resolution, diaphragm and sampling frequency of the FTIR spectrometer were set to 4 cm^-1^, 8 mm and 20 Hz, respectively. The time to acquire an infrared spectrum was approximately 1 s under these parameters. A silver halide FOEW sensor (Fiber Optic ATR Loop Probe, Flexispec^@^ Art Photonics, Berlin, Germany) consisted of a transmission fiber optic with a length of 1000 mm and a spiral FOEW tip with a length of approximately 21 mm. This tip could easily be replaced and connected to the transmission fiber optic when needed. The light beam between the silver halide FOEW sensor and the FTIR spectrometer was connected using a fiber optic coupler (Universal Fiber Probe Coupler, Flexispec^@^ Art Photonics). This coupler could be directly mounted on a pedestal in the sample chamber of the FTIR spectrometer. The silver halide FOEW sensor was then connected to the coupler using two SMA905 interfaces. The IR spectra of the volatile compounds were collected using OPUS 7.0 software (Bruker Ltd.) by connecting a computer (PC) to the FTIR spectrometer with a data cable.

### 2.3 Methods

#### 2.3.1 Feasibility of detecting volatile ethanol in air using an FOEW sensor

Detecting volatile compounds in air using MIR FOEW spectroscopy is challenging (Dong et al., 2019). Therefore, an experiment to verify the feasibility of our proposed method was first carried out. First, the FTIR spectrometer was set to the single measurement mode to acquire the FOEW infrared spectra of the air and headspace volatiles of 10% and 30% ethanol solutions. In this mode, a FOEW infrared spectrum was obtained by averaging 16 spectra. The FTIR spectrometer was then set to the repeated measurement mode to continuously acquire the FOEW infrared spectra alternately between the air and headspace volatiles of the 30% ethanol solution. Three consecutive measurements between air and volatiles were made alternately. The infrared spectra of water and ethanol in the National Institute of Standards and Technology (NIST) Reference Database were used for a comparative analysis with those of air and headspace volatiles. The dynamic response characteristics of the detection system are very important during the on-line measurement of fruit volatiles. Therefore, the absorbance of the ethanol at the 1045 cm^-1^ peak was extracted from the obtained spectra to analyze the response and recovery times of the FOEW sensor.

#### 2.3.2 Quantitation of ethanol in air based on the FOEW spectra

To quantify the concentration of ethanol released from grapes during storage, we acquired the FOEW spectra of standard gas of ethanol at different concentrations in nitrogen using the FTIR spectrometer in the single measurement mode. In this mode, a FOEW infrared spectrum was obtained from the average of 16 spectra. A calibration curve between the absorbance at 1045 cm^-1^ was then extracted from these FOEW spectra and the ethanol concentrations were established based on a chemometrics method. The analytical curves were performed using standard gas of ethanol at 12 different concentrations. Standard gas was analyzed in triplicate by FOEW spectra, and the intensity of characteristic absorption peak for each replica of standard gas were observed and averaged to obtain the calibration curves. We could then quantitatively analyze the concentration of ethanol released from the grapes from this curve.

#### 2.3.3 Analysis of FOEW spectra of grape volatiles and identification of grape spoilage

When detecting the grape volatiles, the FTIR spectrometer was set to the single measurement mode. In this mode, an FOEW infrared spectrum was obtained from the average of 16 spectra. To allow the grapes to decay gradually, five glass bottles containing samples of fresh grapes were stored for 8 d at 22°C and 50% relative humidity. The bottles were sealed for 5 hours from 9 a.m. every day. An infrared background spectrum of the indoor air was acquired as a reference spectrum before opening these bottles. The bottles were then opened in sequence at 2 p.m. every day and immediately placed under the spiral silver halide FOEW sensor to obtain the infrared spectra of the volatile compounds from the grapes. Forty FOEW infrared spectra of the grape volatiles were obtained during the whole experiment. To take account of the strong absorption interference of water in the MIR spectral region (Raichlin and Katzir, 2008), the infrared spectra of the headspace volatiles of deionized water in air were obtained and used as reference spectra for the differential spectral treatment for the grapes. Using the mathematical difference between the absorbance at 1045 cm^-1^ from the ethanol standard gases concentration, the ethanol contained in the grape volatiles was quantified. From the visible light images of the grapes during storage and an analysis of changes in the absorption peak intensity and concentration of ethanol, principal component analysis (PCA) of the infrared spectra of the grape volatiles was used to identify grape spoilage using Unscrambler X software (CAMO Software AS, Oslo, Norway). Partial least squares discriminant analysis (PLS-DA) was used to predict the process of grape spoilage using Matlab (Matlab2019a, MathWorks, Natick, USA), among which the Kennard-Stone sampling algorithm was used to divide the data matrices of grape samples into the calibration and prediction sets and applied using Matlab.

## 3 Results and discussion

### 3.1 Detection of ethanol in air by the MIR FOEW spectrum

**Figure 2:**
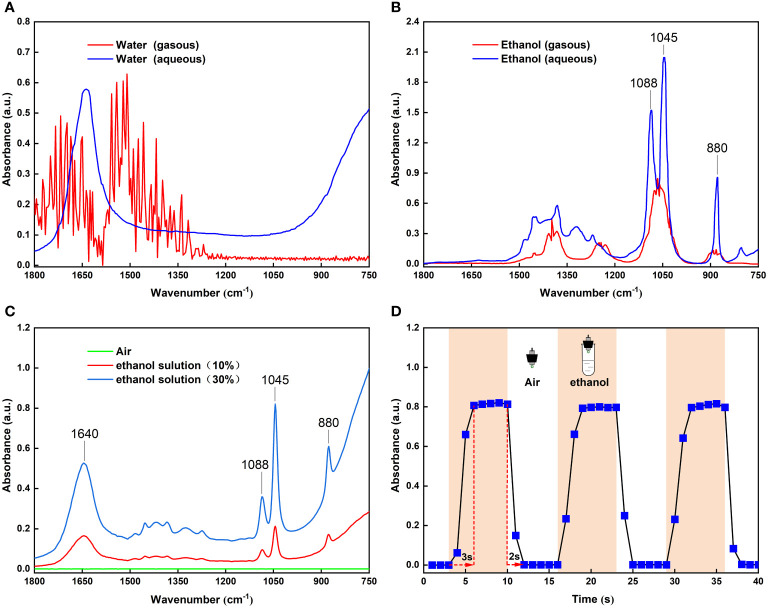
**(A)** Infrared spectra of gaseous and aqueous water from the NIST standard reference database; **(B)** Infrared spectra of gaseous and aqueous ethanol from the NIST standard reference database; **(C)** FOEW infrared spectra of headspace volatiles from 10% and 30% ethanol solutions in test tubes; **(D)** Changes in absorbance at 1045 cm^-1^ peak with time when FOEW sensor was alternately placed in the air and headspace volatiles of the 30% ethanol solution.

In this study, the FOEW spectra of the headspace volatiles of 30 mL 10% and 30% ethanol solutions in plastic tubes were obtained in air (**Figure 2C**). By comparison with the infrared absorption spectra of gaseous and aqueous ethanol in the NIST Reference Database (**Figure 2B**), the absorption peaks of aqueous ethanol at 880, 1045 and 1088 cm^-1^ were clearly detected in our experiments (**Figure 2C**). A similar comparison (**Figure 2A**) also revealed a distinct absorption peak of aqueous water at 1640 cm^-1^, with a strong absorption band from 1000 to 750 cm^-1^ in **Figure 2C**. However, in previous studies on the detection of food volatiles based on a long-path gas cell, the absorption spectra of water and ethanol exhibited gaseous absorption peaks (Dong et al., 2013; Dong et al., 2014b; Jiao et al., 2017), possibly because of the interaction of the water and ethanol molecules surrounding the fiber core surface thus forming aqueous ethanol. Generally, the penetration depth of evanescent field region is very small (Sharma et al., 2019). Over such a small range of action, the ethanol molecules surrounding the core surface and the water volatilized by the ethanol solution formed aqueous ethanol which absorbed the evanescent waves on the core surface of the optical fiber. Therefore, the sensor developed based on FOEW was able to detect volatile compounds in air.

As well as verifying the sensor sensitivity, other performance characteristics such as dynamic response, are very important. To demonstrate the reversibility of the sensor’s response, we conducted time-dependent measurements by repeatedly exposing the sensor to indoor air and the headspace volatiles of the 30% ethanol solution. **Figure 2D** shows the absorbance of ethanol at 1045 cm^-1^ when switching from indoor air to the headspace volatiles of the 30% ethanol solution three times. It can be seen that the absorbance signal returned to the original baseline level, meaning that the sensor exhibited good reversibility. The dynamic response characteristics showed a response time of approximately 3 s and a recovery time of approximately 2 s. These advantages lead to the possibility of its use for detecting ethanol in fruit volatiles *in situ* and on-line, which are urgently demanded in today’s industry (Xia et al., 2016).

### 3.2 Quantitative analysis by the MIR FOEW spectrum

**Figure 3:**
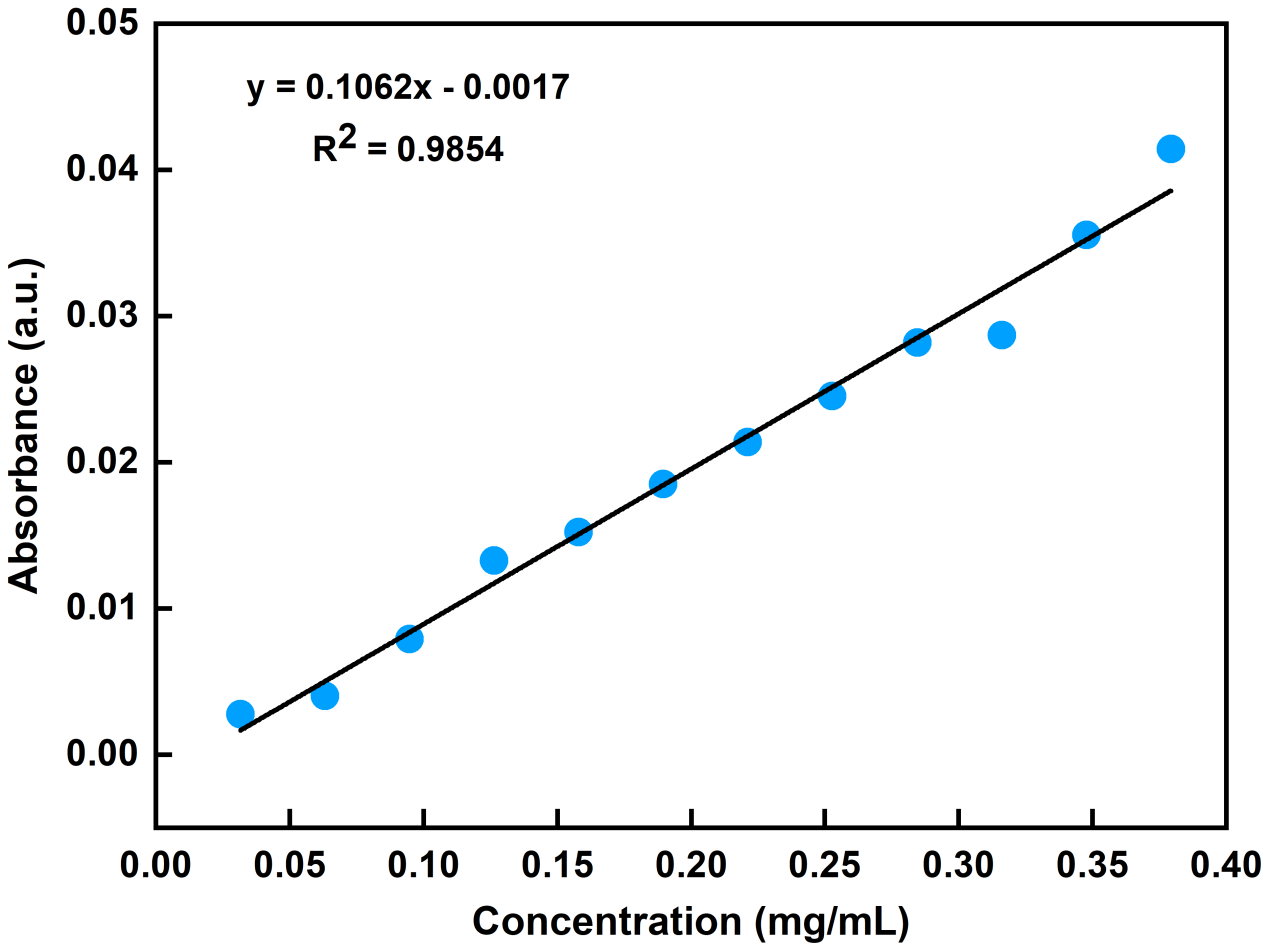
FOEW calibration curve of absorption at 1045 cm^-1^ corresponding to different concentrations of ethanol standard gases.

The absorbance of ethanol increased with increasing concentration of the ethanol standard gases (**Figure 2C**). This indicated the potential for using the FOEW sensor for the quantitative detection of the concentration of ethanol by the absorbance of the ethanol volatiles. To quantify the concentration of ethanol volatiles, we acquired the FOEW spectra of standard gases of ethanol at different concentrations in nitrogen. The relationship between the absorbance of the ethanol volatiles in the glass bottle and the concentrations of ethanol standard gases was then calculated. The absorbance at 1045 cm^-1^ extracted from these FOEW spectra could then be used to determine the concentration of ethanol. The calibration curve analyzed using linear regression (y = 0.1062x - 0.0017) was developed (**Figure 3**), exhibited a good linearity (R^2^ = 0.9854).

### 3.3 Detection of ethanol of grapes in air by the MIR FOEW spectrum

**Figure 4:**
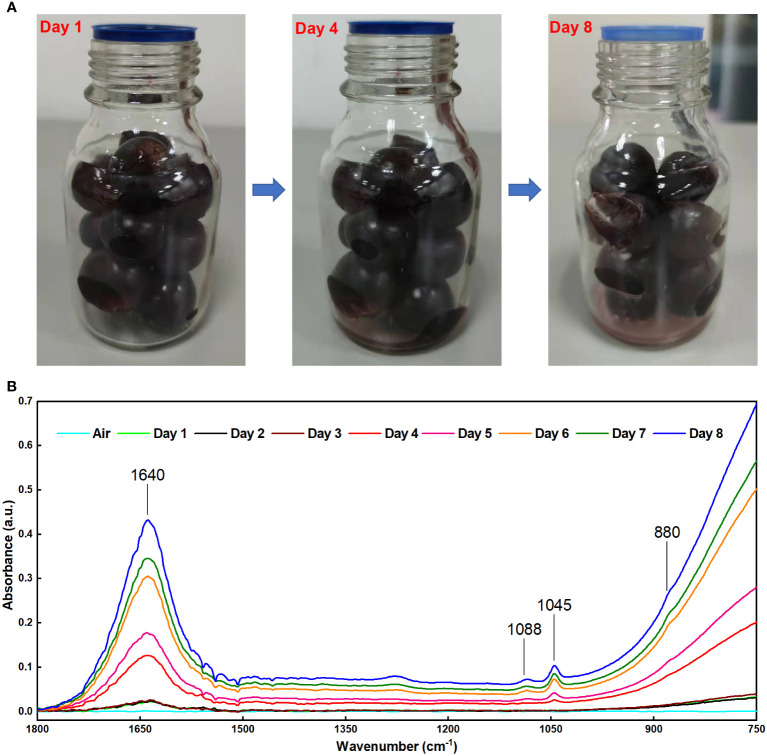
**(A)** Visible light images of grapes at different storage times; **(B)** FOEW infrared absorption spectrum of the grape volatiles at different storage times.

The FOEW infrared spectra of the grape volatiles during storage are shown in **Figure 4B**. In the first three days, we observed the weak absorption peaks of aqueous water at 1640 and 900–750 cm^-1^ in the grape volatiles. **Figure 4A** also showed that there were no changes in the grape skin during the first three days, with the grapes appearing fresh. On the fourth day, a weak absorption peak was detected at 1045 cm^-1^, indicating that there was a small amount of ethanol in the grape volatiles. The water absorbed in the grape volatiles also increased greatly. These trends in the amounts of ethanol and water in the grape volatiles agreed with previous results (Dong et al., 2014a; Ding et al., 2017; Jiao et al., 2019). **Figure 4A** also showed that some slightly white hyphae appeared on the grape surface on the fourth day. This became more apparent on the eighth day, possibly indicating a large change in the physiological activity of the grapes (Ding et al., 2017). On the fourth day and on every day thereafter, the absorbance of aqueous water and ethanol both gradually increased, which was entirely consistent with the previous results (Dong et al., 2014a; Ding et al., 2017; Jiao et al., 2019). This indicated that the grape volatiles contained greater amounts of water and ethanol and that the deterioration of the grapes had increased.

**Figure 5:**
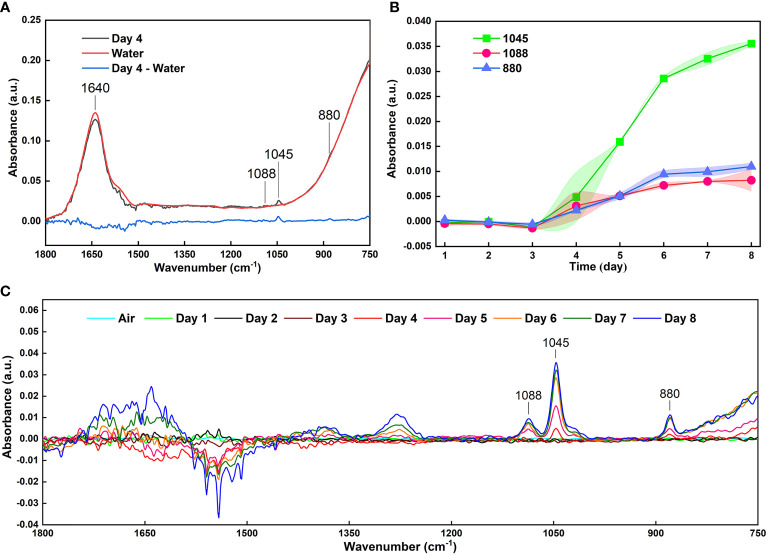
**(A)** Reducing the interference of water absorption by the difference spectra; **(B)** The curves of the absorbance with an error band at absorption peaks of 1088, 1045 and 881 cm^-1^ of 40 infrared spectra of grape volatiles after reducing the interference from water absorption; **(C)** Infrared spectra of grape volatiles after subtracting the interference from water absorption.

In previous studies using methods based on MIR spectroscopy, one of the major difficulties for detecting volatiles released from fruits is the high-water content (Raichlin and Katzir, 2008; Dong et al., 2019). Water has strong absorption in the MIR FOEW (Raichlin and Katzir, 2008). Therefore, it is difficult to measure the absorbance of samples that contain water, a problem confirmed in this study. In the present study, there was serious interference in the detection of ethanol in the range of 900–750 cm^-1^ when a large amount of water was present in the volatiles (**Figure 2C**). This is the reason for the absorption peaks at 880 cm^-1^ showing unobvious changes (**Figure 4B**). To reduce the strong interference due to water absorption, previous studies collected data using deionized water as the background spectrum or used the difference spectroscopy method to mitigate the effects of moisture on the FOEW sensors (Lu et al., 2016; Bančič, et al., 2018; Zhang et al., 2018). In the present study, the infrared spectra of the headspace volatiles of deionized water in air were obtained then used as reference spectra to be subtracted from those of the grape volatiles (**Figure 5A**). In comparison with **Figure 4B**, the characteristic absorption peak at 880 cm^-1^ was clearly present after the difference spectra and showed a similar pattern of change to that at 1045 cm^-1^ (**Figure 5C**). Therefore, the strong interference from water absorption could be effectively reduced and the signal-to-noise of this system could also be increased (Jahromi et al., 2021).

**Figure 6:**
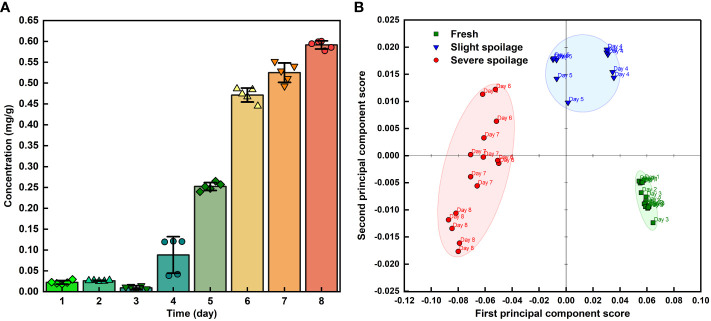
**(A)** The ethanol concentration in volatiles released from grapes during storage; **(B)** PCA analysis of 40 infrared spectra of grape volatiles corresponding to different storage times.

Using this method, 40 sets of spectra from grape volatiles obtained during the 8 days of storage (5 sets of spectra per day) were processed. The absorbances at 1088, 1045 and 881 cm^-1^ were extracted from these spectra. The curves of those absorbances with error bands are shown in **Figure 5B**. The overall pattern of change of the absorbance for the three peaks was basically the same. The slope of these curves began to increase during the 3rd and 4th days, indicating that the grapes had begun to emit ethanol during this period. The maximum slopes were observed between the 4th and 6th days of storage, implying that the rate of ethanol release increased as the grapes deteriorated, which agreed with the previous results (Dong et al., 2014a; Ding et al., 2017; Jiao et al., 2019). The decrease in the slope between the 6th and 8th days was caused by the loss of water from the grapes. This might have arisen from poor physiological activity in the grapes or the dissolution of ethanol in the juice at the bottom of the bottle as shown in the visible light image of the grape on the 8th day (**Figure 4A**). The greatest error band appeared between the 3th and 5th days, particularly on the 4th day, indicating that the rate of ethanol release was unstable during this period. This could therefore suggest that during the transition period from fresh to spoiled grapes, the physiological activity of the grapes or of the microorganisms was highly variable, which was consistent with our previous study (Dong et al., 2014a). Subsequently, in combination with quantification from the FOEW sensor calibration curve of the absorption at 1045 cm^-1^, the ethanol concentration in volatiles released from the grapes were analyzed quantitatively (**Figure 6A**).

### 3.4 Analyzing spoilage of grapes by the MIR FOEW spectrum

Grapes are one of the most perishable fruits (Shen and Yang, 2017), with post-harvest losses caused by decay and from water loss after harvest and during storage (Leng et al., 2022). Previous study found that ‘Kyoho’ grapes began to spoil at the room temperature at approximately the 4th day and defined three freshness categories: “Fresh,” “Slight spoilage,” and “Severe spoilage” (Dong et al., 2014a; Ding et al., 2017; Jiao et al., 2019). After observing the visible light images of the grapes in **Figure 4A** and the analysis of the infrared spectra of the grape volatiles, we suggest that the grapes were fresh between the 1st and 3rd days of storage, with the FOEW sensor not detecting obvious ethanol volatiles during this period; slight spoilage between the 4th and 5th days, at which point the sensor detected between 0.088 and 0.252 mg ethanol per g of grape volatiles; then severe spoilage after 6th to 8th days, at which time the sensor detected between 0.471 and 0.591 mg ethanol per g of grape volatiles. The data obtained from the ethanol concentration of grape volatiles provided crucial criteria for distinguishing the three stages of grape spoilage.

Principal component analysis (PCA) can be performed as an unsupervised classification method to visualize the resemblances and differences between different measurements. After subtracting the interference from water absorption, 40 infrared spectra of grape volatiles corresponding to different storage times were used for PCA analysis (**Figure 6B**). PCA analysis used data from the 1800–750 cm^-1^ wavelength bands, with contributions of 92% and 4% from PC1 and PC2, respectively. The grape samples were separated along the first PC which described 92% of the peak variation and showed three defined groups. Along the PC1 axis, the storage times of grapes showed obvious clustering at 1-3, 4-5 and 6-8 days of storage. The analysis of the infrared spectra of the grape volatiles suggested that the grapes were fresh between the 1st and 3rd days of storage, slightly spoiled at between the 4th and 5th days then severely spoiled after 6 to 8 days of storage. This was basically consistent with the PCA analysis, indicating that this method based on the FOEW sensor can be used to accurately detect grape spoilage by monitoring the spoilage odors from the volatile substances when the grapes were stored.

The results of PCA cluster analysis served as a reference for establishing a qualitative model based on FOEW spectral data sets combined with a PLS-DA algorithm. The data matrices produced for the grape samples during storage were divided into the calibration and prediction sets in a ratio of 3–1, using the Kennard-Stone method. Of the 40 spectral data from grape samples used for classification, 30 samples were in the calibration set, and 10 samples in the validation set. The accuracies of the calibration and validation sets both were 100%, which indicated that the model had good stability and predictability. It therefore appears that FOEW spectra could reliably predict the process of grape spoilage.

## 4 Conclusions

This study has used a method based on the FOEW sensor and FTIR spectrometer to detect grape volatiles in air *in situ* and has identified a marker volatile compound (ethanol) that indicates grape spoilage. When combined with chemometric analysis, grape spoilage could be accurately identified. Compared with the method used in previous study based on the long-path gas cell, this method uses a miniature FOEW sensor placed directly near the fruit without the need to sample the fruit itself. This not only eliminates the potential pollution of the gas source used for injection into the FTIR spectrometer, but also achieves a practical method for detecting the volatile compounds released by fruit *in situ*. Fiber optic sensing technology is not affected by electromagnetic interference and can be distributed over long distances for on-line measurement. The proposed technique is expected to provide a new method for the long-distance, *in situ* and on-line detection of the volatile compounds released from fruit. This will allow the rapid identification of fruit deterioration. In addition, the sensitivity and specificity of the FOEW sensor could be greatly improved by coating a thin film (nanoparticles, metal-organic frameworks and so on) on the fiber core to accumulate the volatiles, which will be applied in our future work.

## Data availability statement

The raw data supporting the conclusions of this article will be made available by the authors, without undue reservation.

## Author contributions

YZ: conceptualization, drafted the manuscript and revised it. LJ: contributed to the funding acquisition, and supervision of the study and made substantial contributions to the revision of the manuscript. YG, RG, KW, and QZ: some experiments, performed the data acquisition and analysis. DD: conceptualization, writing the manuscript, read, and agreed to the submitted version. All authors contributed to the article and approved the submitted version.

## Funding

This research was funded by the National Natural Science Foundation of China (31972148, 32101609), Financial special project of Beijing Academy of Agriculture and Forestry Sciences (CZZJ202204), and Beijing Innovation Consortium of Agriculture Research System (BAIC08-2022).

## Acknowledgments

The authors are grateful to reviewers for their valuable comments and suggestions.

## Conflict of interest

The authors declare that the research was conducted in the absence of any commercial or financial relationships that could be construed as a potential conflict of interest.

## Publisher’s note

All claims expressed in this article are solely those of the authors and do not necessarily represent those of their affiliated organizations, or those of the publisher, the editors and the reviewers. Any product that may be evaluated in this article, or claim that may be made by its manufacturer, is not guaranteed or endorsed by the publisher.
